# Adipose Extracellular Vesicles: Messengers From and to Macrophages in Regulating Immunometabolic Homeostasis or Disorders

**DOI:** 10.3389/fimmu.2021.666344

**Published:** 2021-05-24

**Authors:** Zixin Zhou, Yan Tao, Hui Zhao, Qun Wang

**Affiliations:** ^1^ Key Laboratory of Infection and Immunity of Shandong Province, Department of Immunology, School of Basic Medical Sciences, Cheeloo College of Medicine, Shandong University, Jinan, China; ^2^ Department of Clinical Laboratory, The Second Hospital, Cheeloo College of Medicine, Shandong University, Jinan, China

**Keywords:** extracellular vesicle, macrophage, exosome, immunometabolism, adipose-derived stem cell, obesity, adipocyte, adipose tissue

## Abstract

Adipose tissue is comprised of heterogenous cell populations that regulate both energy metabolism and immune reactions. Macrophages play critical roles in regulating immunometabolic homeostasis or disorders through cooperation with adipocytes, adipose tissue-derived stem cells (ADSCs) or other cells in adipose tissue. Extracellular vesicles (EVs) are recently recognized as efficient messengers for intercellular communication. Emerging evidences have demonstrated that adipose EVs are actively involved in the mutual interactions of macrophages, adipocytes and ADSCs, which produce considerable influences on immunometabolism under healthy or obese conditions. Here, we will elaborate the production and the characteristics of adipose EVs that are related to macrophages under different metabolic demands or stresses, whilst discuss the roles of these EVs in regulating local or systemic immunometabolic homeostasis or disorders in the context of adipocyte-macrophage dialogue and ADSC-macrophage interaction. Particularly, we provide a profile of dynamic adipose microenvironments based on macrophages. Adipose EVs act as the messengers between ADSCs and macrophages to maintain the balance of metabolism and immunity, while drive a vicious cycle between hypertrophic adipocytes and inflammatory macrophages to cause immunometabolic imbalance. This review may provide valuable information about the physio- or pathological roles of adipose EVs and the application of adipose EVs in the diagnosis and treatment of metabolic diseases.

## Introduction

Adipose tissue is the major metabolic organ that regulates glycolipid metabolism and energy balance. There are two types of adipose tissues, which perform distinct functions in energy regulation. White adipose tissue (WAT) stores surplus energy in the form of triglycerides, whereas brown adipose tissue (BAT) dissipates energy through thermogenesis to maintain body temperature. The Imbalance between energy intake and consumption may lead to obesity manifested by excessive fat accumulation and pathological expansion of WAT (1–3). This disturbance is often accompanied by WAT inflammation, characterized by infiltration and activation of proinflammatory immune cells such as macrophages and T cells, as well as high levels of proinflammatory cytokines. The chronic inflammation caused by WAT dysfunction leads to insulin resistance in liver, muscle, adipose tissue, and result in metabolic abnormalities such as hyperglycemia, hypertension and dyslipidemia, thereby linking obesity with type 2 diabetes and cardiovascular diseases (4–7). So, WAT is the main site for local or systemic immunometabolic regulation under both healthy and pathological conditions.

Tissue immunometabolism refers to the connection of immunity and metabolism in metabolic tissues including liver, muscle, pancreas, and adipose tissue. The regulation of immunometabolism in these tissues relies on the mutual interactions among tissue parenchymal cells, stromal cells, and immune cells. These cellular interactions develop adaptation to each other to maintain immunometabolic homeostasis and normal physiological functions of metabolic organs under healthy condition or in response to acute metabolic demands. In case of obesity or chronic metabolic stresses, the infiltration and activation of immune cells may impinge the actions of metabolic hormones like insulin on parenchymal cells, and further impair the glycolipid metabolism in metabolic organs, eventually resulting in tissue maladaptation and metabolic disorder clusters. For example, lean WAT contains large amounts of immunomodulatory cells such as alternatively activated macrophages, regulatory T cells, type 2 innate lymphoid cells, which cooperate with stromal cells to maintain tissue homeostasis and support physiological functions of adipocytes. In contrast, obese WAT is characterized by accumulation and activation of immune cells including inflammatory macrophages, effector CD4^+^ and CD8^+^ T cells (mainly Th1 and CTL), which induce tissue inflammation and cause insulin resistance in adipocytes, hepatocytes and myocytes, thereby contributing to tissue dysfunction and associated metabolic complications (1, 8–10). In adipose tissue, adipocytes, adipose-derived stem cells (ADSCs) and macrophages act as the main players of parenchymal cells, stromal cells, and immune cells, respectively, which actively participate in immunometabolic regulation. Kinds of soluble factors, comprising adipokines, cytokines, growth factors and fatty acids, are involved in the intercellular communication to regulate immunity and metabolism, which have been well reviewed elsewhere and will not be detailed here (1, 5, 8, 11, 12). Extracellular vesicles (EVs), as another form of soluble factors, are recently recognized as the important modulators affecting neighboring or distant cells. As membrane-coated vesicles, EVs carry and transport various of bioactive proteins, lipids, or nucleic acids from donor cells into recipient cells, thereby affecting their biological characteristics and functions (13–17). Emerging data have shown the critical roles of EVs in regulating immunometabolism in metabolic tissues, particularly in adipose tissue. Herein, we will summarize the production and characteristics of EVs from WAT and their roles in maintaining or disrupting immunometabolic homeostasis in the context of adipocyte-macrophage dialogue and ADSC-macrophage interaction.

## Cells in Adipose Tissue Related to Immunometabolism

In the 1960s, Rodbell pioneered the study on individual cell components of adipose tissue, and showed that hormones produced similar metabolic effects on isolated fat cells and fat tissue (18). Thus, to some extent, fat cells could be used as substitute for adipose tissue in particular studies. In fact, fat cells only account for part of the total cells in adipose tissue. Aside from adipocytes, a cluster of stromal vascular fractions, comprised of ADSCs, immune cells, endothelial cells, and other cell components, are found in WAT (19–22). Among them, both innate and adaptive immune cells such as macrophages, natural killer cells, T cells and B cells contribute to the formation of dynamic immune microenvironments with the changing metabolic status, wherein various immune cells communicate with adipocytes, ADSCs or other cells (23–26). Here, we will discuss the adipocytes, ADSCs and macrophages in detail.

### Adipocytes

As the parenchymal cells of adipose tissue, adipocytes not only function as critical regulators for energy metabolism, but also serve as endocrine modulators involved in various physio- or pathological processes like appetite control and immune response (5, 11, 27–30). White adipocytes, mainly present in WAT throughout the body, contain large and unilocular lipid droplets to store energy in the form of triglycerides. In contrast, brown adipocytes, mainly distributed in BAT in the scapular area and neck, contain small and multilocular lipid droplets together with abundant mitochondria that contribute to energy dissipation in the form of heat (3, 31–33). In addition, beige adipocytes, as brown-like adipocytes induced by cold stimuli or high-fat diet (HFD) challenge, also contribute to energy consumption *via* heat production (34, 35). The three types of adipocytes function differently and cooperate with each other in response to various metabolic demands or stresses. For instance, white adipocytes contribute to WAT expansion *via* hyperplasia and hypertropia during obesity, whereas the activation of brown adipocytes and the induction of beige adipocytes reduce obesity and improve insulin sensitivity. White adipocytes play important roles in regulating glycolipid metabolism and energy balance through storing excess energy and supplying it when needed. Large amounts of secretary factors from adipocytes, such as leptin, adiponectin, cytokines, fatty acids and EVs, are involved in these processes, which mediate the communication of adipocytes with other cells inside or outside adipose tissue (5, 11). Besides their functions in energy storage and endocrine, white adipocytes have recently been recognized to regulate both innate and adaptive immunity in adipose tissue, such as recruiting and activating macrophages, presenting antigens to invariant natural killer T cells or CD4^+^ T cells (25, 36–40). Thus, adipocytes may be the major cell components in regulating WAT immunometabolism. In this review, we mainly focus on the EVs related to white adipocytes in WAT.

### ADSCs

ADSCs are the dominant stromal cells in adipose tissue that serve as progenitors responsible for the regeneration and replenish of adipocytes. ADSCs have great potentials for homing and self-renewal, as well as strong capacity for multiple differentiation toward adipocytes, chondrocytes, and muscle cells. Therefore, ADSCs are currently recognized as promising therapeutic candidates for tissue repair and regeneration (7, 8). The proliferation and adipogenesis of ADSCs are essential for the maintenance of metabolic homeostasis particularly in adipose tissue, as supported by our previous study showing distinct metabolic influences on WAT homeostasis by ADSCs from different anatomic locations (41, 42). Notably, human or mouse ADSCs showed strong capacity for immunomodulation through actively participating in both innate and adaptive immunity, such as promoting macrophage polarization toward anti-inflammatory phenotypes or inducing regulatory T cell differentiation. These immunomodulatory effects of ADSCs were perfectly reflected in the treatment of several inflammatory diseases like colitis and sepsis in animal models (43–46). While for metabolic inflammation, ADSCs also brought desirable effects on the improvements of immune microenvironments in adipose tissues or the attenuation of inflammation and tissue damages in animal models with obesity or type 2 diabetes (47–50).

### Macrophages

The great interest on adipose tissue-resident macrophages (ATMs) is largely ignited by the discovery that obesity induced the infiltration and activation of macrophages in WAT to elicit inflammation and insulin resistance (51, 52). Monocyte chemoattractant protein-1 (MCP-1) is recognized as the main contributor to the increase of ATMs in obese mice. Besides, fatty acids released by necrotic fat cells are also phagocytic stimuli for ATMs, which promote their infiltration around necrotic cells to form crown like structures (12, 53, 54). In fact, macrophages are critical resident cells in WAT, which not only mediate WAT inflammation and metabolic disorders under obese condition, but also participate in the maintenance of WAT immunometabolic homeostasis under lean condition. Macrophages mediate divergent effects on immunometabolism depending on their different phenotypes. Based on different stimuli, macrophages can be classified into classically activated M1 and alternatively activated M2 subsets. M1 macrophages activated by lipopolysaccharide (LPS) plus interferon (IFN)-γ release proinflammatory cytokines tumor necrosis factor (TNF)-α, interleukin (IL)-1, IL-6 and so on; while M2 macrophages activated by IL-4 and IL-13 release IL-10 and arginase-1 to play an anti-inflammatory effect. Different from the response of macrophages to *in vitro* stimulation, ATMs *in vivo* display more complexity and flexibility in gene profiles, phenotypes, and functions. Though many researchers tend to classify ATMs into M1-like and M2-like macrophages, we will specify their characteristic phenotypes in this review unless these characters are not indicated in these studies. As evidenced by the findings from Lumeng and Fujisaka et al., two types of ATMs dominate the regulation of WAT immunometabolism in different metabolic status. Under lean condition, CD206^+^ ATMs are dominant in epidydimal WAT characterized by M2 phenotypes (high expression of Ym1, arginase 1 and IL-10), which facilitate immunometabolic homeostasis in WAT. While diet-induced obesity induces a population of CD11c^+^ ATMs characterized by M1 phenotypes (high expression of iNOS and TNF-α) that drive immunometabolic disorders in WAT (55–57). Once obesity induces the phenotype switch of ATMs, proinflammatory cytokines secreted by M1 ATMs, particularly TNF-α, IL-1 and IL-6, play a direct role in promoting insulin resistance and exacerbating local or systemic metabolic dysfunction. On the contrary, IL-10 produced by M2 ATMs can relieve TNF-α-induced insulin resistance (27, 55, 58, 59). Besides aforementioned ATMs, other distinct populations of ATMs have recently been identified to exert both immune and metabolic functions and will not be detailed here (60).

## Adipose EVS in Regulating Immunometabolism

**Figure 1:**
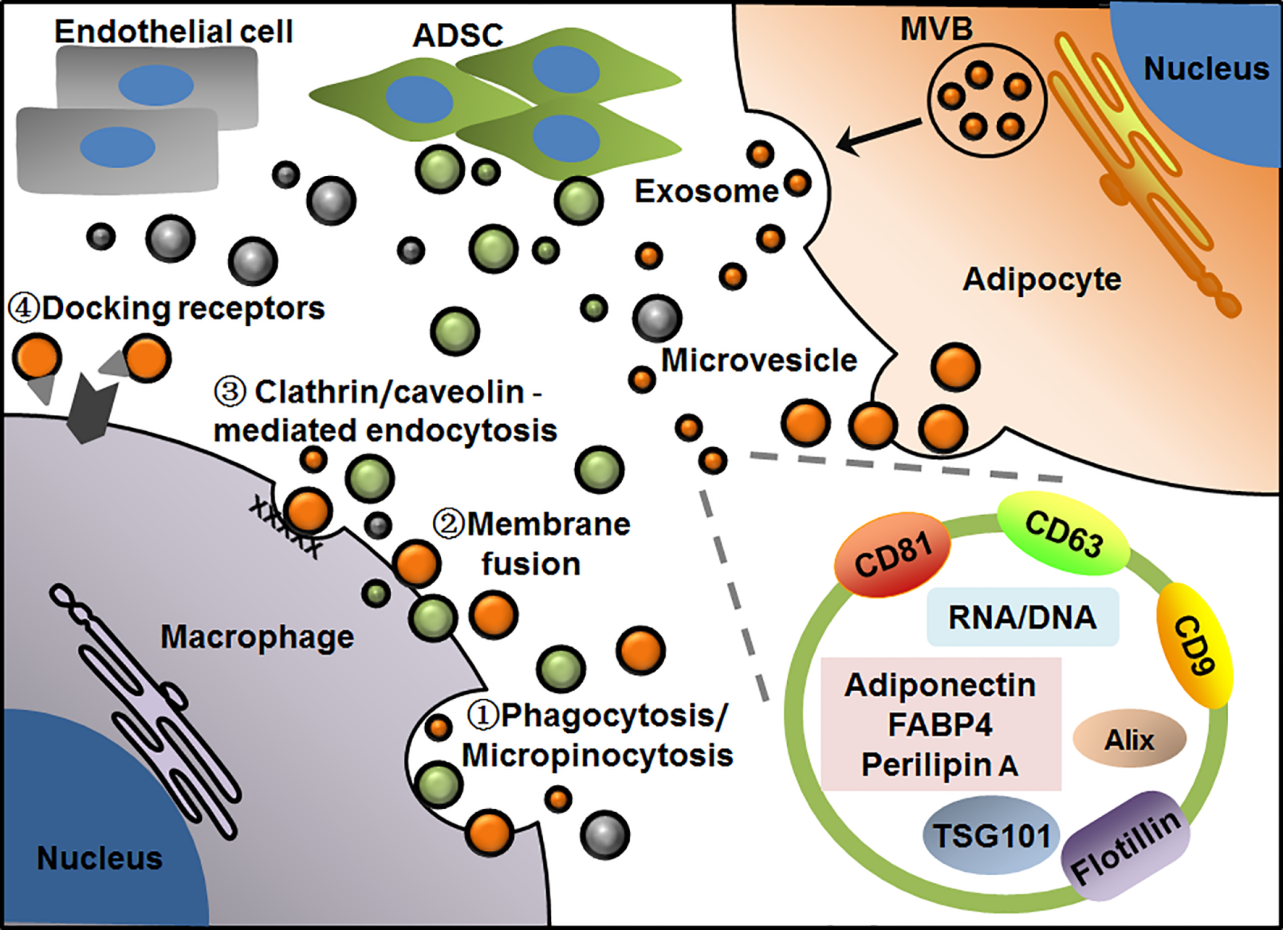
Patterns of EV-mediated communication of adipocytes with macrophages in adipose tissue. Adipocytes release microvesicles and exosomes through outward budding of plasma membrane or exocytosis *via* MVB pathway, respectively. Macrophages take up EVs by phagocytosis/micropinocytosis①, direct membrane fusion②, clathrin/caveolin -mediated endocytosis③ or binding with particular docking receptors④. Adipocyte-derived exosomes display general markers such as CD81, CD63, CD9, Alix, TSG101 and flotillin, as well as several cell-specific molecules such as adiponectin, FABP4 and perilipin A. EVs from adipocyte, ADSC or endothelial cell are presented in orange, green and gray, respectively.

EVs surrounded by bilayer membranes are produced by the cells and released into extracellular space. Because of their capacity for carrying and delivering various cargos to neighboring or distant recipient cells in an easy and safe way, EVs are recently recognized as the efficient messengers between cells even organs. EVs can be divided into microvesicles and exosomes according to sizes and biogenesis. Microvesicles, sometimes referred to as microparticles or ectosomes in earlier studies, have a larger size of about 100-1000 nm in diameter, which are usually released from plasma membrane into extracellular space through outward budding. So, the molecule compositions of microvesicles are largely dependent on their cell sources and may vary a lot with cell types. In contrast, exosomes are around 30-150 nm in diameter, which are generated from the endosome membrane by inward budding to form intraluminal vesicles inside multivesicular bodies (MVBs). Exosomes are released by the fusion of MVBs with plasma membrane and exocytosis (13, 61, 62). Thus, exosomes from different cell types usually possess some common proteins involved in exosome biogenesis and release, such as Alix, TSG101 and several tetraspanins. These molecules have been well recognized as exosome markers, among them CD9, CD63 and CD81 are commonly used as surface markers. Of note, various bioactive proteins, lipids, and nuclear acids carried by EVs contribute to their heterogeneity and complexity in molecule compositions and functional diversity. Kinds of proteins including membrane proteins, cytosolic and nuclear proteins can be found in EVs. Due to phospholipid bilayer and lipid rafts embedded within the membranes, EVs contain plentiful lipids such as sphingomyelin, cholesterol and ceramide. In addition, different kinds of DNA and RNA including mRNA and noncoding RNA are also enclosed in EVs. As for the uptake of EVs by recipient cells, several different pathways have been proposed, which include phagocytosis or micropinocytosis, direct membrane fusion, clathrin or caveolin-mediated endocytosis, as well as lipid raft-mediated endocytosis. While other particular docking receptors for EV binding remain to be uncovered. These uptake modes of EVs may depend on the types and physiological states of recipient cells (63–67) (**Figure 1**).

### Adipose EVs

In WAT, many kinds of cells including aforementioned adipocytes, ADSCs and macrophages have been found to produce EVs to regulate local or systemic immunity and metabolism. As such, EVs act as the pivotal messengers for intercellular communication inside or outside adipose tissue. To date, various profiles and functions of adipose EVs have been revealed by different groups. Based on these findings, some characteristic markers have been found in adipose EVs, which are helpful for us to understand their exact cell sources and functions in circulation or tissues. In recent studies, adiponectin and fatty acid binding protein 4 (FABP4) were usually considered as the specific markers of EVs from adipocytes (68–70), while perilipin A was identified as a biomarker of stressed adipocytes in case of obesity in both human and mice (71). In addition, it has recently been found that CD31 specific for endothelial cells was highly enriched in EVs from primary endothelial cells of the mice, which may be added to the list of cell-specific markers of adipose EVs (72). As for EVs from other cell components, most of the studies used common exosome markers, whereas few studies reported their specific markers related to cell types. For instance, CD9, CD63, TSG101 were usually used to identify exosomes isolated from both primary ADSCs and macrophages regardless of cell sources (73) (**Figure 1**).

In terms of adipose-related EVs, several earlier studies put more attention on the alteration and overall effects of these EVs upon obesity rather than their specific sources and functions. In these studies, EVs were isolated from adipose tissue explants or plasma regardless of their cell sources (74–76), so it is relatively difficult to determine their exact cell-to-cell pathways and related regulatory mechanisms. Besides the direct influences on WAT functions (discussed later), adipose EVs also influence remote metabolic tissues such as liver and muscle by regulating local or systemic immune microenvironments. Two studies from adolescents demonstrated that exosomes with specific miRNA profile from obese visceral fat targeted TGF-β signaling pathways, which were possibly associated with inflammation and fibrosis in end-organ caused by obesity (75, 77). While another human study revealed that EVs from obese visceral fat impaired the insulin action in HepG2 hepatocytes, possibly related to their high loading of MCP-1, IL-6 and macrophage migration inhibitory factor (MIF) (74). However, there was no evidence for the direct effects of these EV miRNA or cytokines on related signaling pathways in target tissues or cells. Although accumulating data have demonstrated the package of various cytokines in EVs, the precise functions of these EV-associated cytokines remain largely unknown, possibly due to their different action modes from free soluble cytokines. Free soluble cytokines act on recipient cells through binding their specific receptors on plasma membranes to mediate relevant signaling pathways. While EV-packaged cytokines may cause more complicated effects in recipient cells, as they can be taken up through different pathways including phagocytosis, pinocytosis, or receptor-mediated endocytosis (64, 78–80). An earlier study showed that the membrane form of TNF-α carried by exosomes could activate its classical NF-κB signaling pathway. Later, Fitzgerald and colleagues revealed that cytokines could be bounded to EV surface or encapsulated inside EVs (78, 79). Thus, there is still the possibility that adipose EV-associated cytokines activate conventional signaling pathways in recipient cells. As the production of cytokines from the cells in soluble or EV-associated forms may depend on different cell types, stimulus factors and activation status (79–81), it will be more complicated to clarify the fine-tune regulation of adipose EVs on remote metabolic organs. With respect to the regulation of adipose EVs in WAT, emerging studies have demonstrated the precise cell-to-cell crosstalk, which cover the various interactions between adipocytes, stromal cells, endothelial cells, and immune cells. For example, Scherer’s group recently revealed that epithelial cells communicated with adipocytes through small EVs (sEVs) in WAT. Using adipocyte-specific caveolin 1 (cav1) knockout mice and a series of *in vivo* and *in vitro* tracking techniques, they demonstrated that sEVs mediated the trafficking of cav1, a membrane-bound protein abundant in adipocytes and endothelial cells, from neighboring endothelial cells to adipocytes. More interesting, this EV transfer was regulated by different metabolic state *in vivo*, which was increased by fasting but returned to basal levels when feeding. Particularly, the production of EVs from the endothelial cells of obese WAT was almost absent, indicating the importance of these EVs in metabolic homeostasis (72).

### Adipose EVs in Adipocyte-Macrophage Dialogue

As critical parenchymal cells and immune cells of WAT, adipocytes and macrophages cooperate with each other to regulate local or systemic immunometabolic homeostasis or disorders (37, 38, 59, 82). As mentioned earlier, ATMs characterized by CD206^+^ M2 phenotypes are abundant in lean adipose tissue, while obesity induces the infiltration and activation of macrophages with CD11c^+^ M1 phenotypes. This phenotype switch of macrophages is currently believed to be caused by hypertrophic or apoptotic adipocytes in obese adipose tissue, which in turn impairs the insulin action in adipocytes (55, 59). The interaction between adipocytes and macrophages can be mediated by multiple ways such as adipocyte-derived free fatty acids and macrophage-derived cytokines, whilst EVs are emerging as the important intercellular messengers to exert functions during this process.

#### Actions of Adipocyte-Derived EVs on Macrophages

It has been demonstrated that both human and mice adipocytes could release EVs that were detectable in circulation. So, adipocytes are the important sources of EVs that influence the microenvironments inside even outside adipose tissue. Upon metabolic stresses like obesity, the production of adipocyte-derived EVs was increased whilst their cargos were changed (71, 83–86). To some extent, these alterations in adipocyte EVs may serve as the indicators of adipose tissue health even biomarkers of metabolic disorders. More importantly, these EVs are actively involved in regulating immunometabolic homeostasis or disorders through acting on nearby or distant target cells including adipocytes, endothelial cells, immune cells even neuron (87–89). Regarding the regulation of adipocyte-derived EVs on immune cells, most of the studies focus on macrophages rather than other cell types. Adipocytes release EVs to act on either monocytes or macrophages, thus producing regulatory effects on both immunity and metabolism. Initially, Deng et al. showed that exosome-like vesicles from visceral fat of obese mice could be taken up by blood monocytes, and then promoted their differentiation into macrophages and activation characterized by TNF-α and IL-6 secretion. Transfusion of these exosome-like vesicles from obese fat significantly impaired the insulin sensitivity of lean mice, which was dependent on toll-like receptor (TLR) 4 pathway. Through *in vitro* experiments, exosomal retinol binding protein 4 (RBP4) was verified to be responsible for the inflammatory activation of macrophages (90). This study raised the possibility that EVs from hypertrophic adipocytes might promote ATM activation and cause obesity-associated inflammation and insulin resistance. Additionally, Renovato-Martins et al. demonstrated that microparticles (with similar sizes to microvesicles) from human obese omental fat could upregulate the expression of CD16 and CCR5 on human monocytes and increased their migration capacity. In particular, they found that these microparticles from obese fat carried abundant TLR8 compared to those from lean fat, which could be transferred into monocytes and induced the expression of CD16 (91). These observations provided evidences for macrophage migration and activation mediated by EVs from obese fat, which may explain at least in part the contribution of these EVs to ATM infiltration and activation in obese WAT. Considering these EVs were isolated from WAT explants, their exact cell sources remained to be confirmed though the circulating EVs from adipocytes were increased in response to obesity. In recent years, accumulating evidences have demonstrated the direct regulation of adipocyte-derived EVs on macrophage functions including differentiation, migration and polarization. Several studies showed that EVs from mouse or human adipocytes not only induced the migration of primary monocytes and macrophages, but also promoted their differentiation into ATM phenotypes, though EVs with different sizes might target to different pathways in recipient cells (70, 92). Several proteins like MIF, macrophage-colony stimulating factor (M-CSF) and TNF-α have been identified in EVs from *in vitro*-differentiated human adipocytes, which might contribute to the proinflammatory phenotypes of macrophages, and these macrophages in turn impaired the insulin action on adipocytes (70), suggesting that EVs could drive a reciprocal interaction between adipocytes and macrophages. Using primary adipocytes from obese mice, Tamara et al. demonstrated that EVs from pathologically hypertrophic adipocytes with distinct protein profile not only promoted macrophage inflammation by elevating TNF-α and IL-6, but also induced adipocyte differentiation and impaired insulin action of heathy adipocytes (93). Since these observations were obtained from *in vitro* culture system, the influences of *in vivo* microenvironments and EV-associated cytokines need to be considered and determined. Notably, a recent study provided an insight into the regulation of adipocytes on ATM polarization *via* exosomes, in which mature adipocytes secreted exosomal miRNA (miR)-34a into macrophages and inhibited CD206^+^ M2 polarization by downregulating Krüppel-like factor 4. In line with the detrimental roles of miR-34a in obesity-associated metabolic dysregulation, these observations may provide an explanation for adipose tissue inflammation mediated by adipocyte-ATM crosstalk *via* exosomal miR-34a in obese mice (94). In a slightly different way, the upregulation of miR-155 in adipocyte-derived microvesicles from obese mice, as shown in another study, contributed to M1 polarization depending on the activation of signal transducer and activator of transcription (Stat) 1, whilst these microvesicles also elicited a significant decline of CD206^+^ M2 percentages in bone marrow-derived macrophages (95). These observations defined EVs as the transporters of specific miRNA from adipocytes to macrophages under obese condition, which mediated macrophage polarization through different ways, probably due to their different types and contents (**Table 1**). Considering the high heterogeneity of EV contents, the combined effects of these EV miRNA cannot be excluded.

**Table 1:** EVs involved in adipocyte-macrophage dialogue and ADSC-macrophage interaction.

Source	EVs	Contents	Target	Function	Reference
**VAT from obese mice** **(adipocytes)?**	Exosome-like vesicles	RBP4	Monocytes Macrophages	Promote differentiation of monocytes into macrophages Promote inflammatory activation of macrophages Induce insulin resistance in mice	Deng et al. Diabetes
**Stressed 3T3-L1 adipocytes**	Microparticles	–	Macrophages	Mediate attraction of macrophages *in vitro* and *in vivo*	Eguchi, A., et al. PLoS One
**Human *in vitro* differentiated adipocytes**	EVs	RBP4, TNF-α, MIF	Monocytes	Differentiate monocytes into macrophages with ATM characteristics (pro- and anti-inflammatory phenotypes)	Kranendonk, et al. Obesity
**Mouse hypertrophied adipocytes**	EVs	–	RAW264.7 macrophages	Promote inflammation of macrophages	Tamara, C. et al. Int J Mol
**Mature adipocytes from** **VAT of obese mice**	Exosomes	MiR-34a	ATMs, BMDMs	Suppress the macrophages polarization toward M2 *in vitro*	Pan, Y. et al. J Clin Invest
**Primary adipocytes from VAT of obese mice**	Microvesicle	MiR-155	BMDMs	Promote M1 macrophage activation	Zhang, et al. J Mol Cell Biol,
**Mouse adipocyte from VAT**	Exosomes	Triglyceride	Bone marrow precursors, ATMs	Induce differentiation of bone marrow progenitors into ATM-like cells	Flaherty, et al. Science
**VAT of obese mouse (adipocytes)?**	Exosomes	–	RAW264.7	Promote formation of macrophage foam cell formation Promote macrophage polarization into M1 phenotypes Exacerbate atherosclerosis in apolipoprotein E–deficient mice	Xie, Z. et al. J Am Heart Assoc
**THP-1 differentiated- M1 macrophages (induced by LPS plus IFN-γ)**	Macrovesicles	–	Human adipocytes	Induce insulin resistance in human adipocytes	Zhang, Y., et al. Nutr Metab
**THP-1-differentiated macrophages (induced by LPS)**	Exosomes	Specific miRNA	Human adipocytes	Change the expression of genes related to inflammation in adipocytes	De Silva, et al. J Physiol Biochem
**High glucose-induced RAW264.7 macrophage**	Exosomes	MiR-210	3T3-L1 adipocytes	Impair glucose uptake and mitochondrial activity in adipocytes	Tian, F., et al. Journal of Diabetes Research
**ATMs from Obese mice**	Exosomes	MiR-155	Mouse adipocytes	Impair insulin action and glucose uptake in adipocytes Cause insulin resistance in lean mice	Ying, W. et al. Cell
**ATMs from lean mice**	Exosomes	–	Mouse adipocytes	Increase insulin action and glucose uptake in adipocytes Improve insulin sensitivity in obese mice	Ying, W., et al. Cell
**Obese ATMs**	Exosomes	MiR-29a	Adipocytes	Impair insulin action in adipocytes Impair insulin sensitivity in lean mice	Liu, T, et al. Biochem Biophys Res Commun
**Lean ADSCs**	Exosomes	Stat3	Macrophages ATMs	Promote macrophage polarization into M2 phenotypes Induce WAT beiging and improve insulin sensitivity in HFD-fed mice	Zhao, H., et al. Diabetes

VAT, Visceral adipose tissue; BMDM, Bone marrow derived macrophage.

Aside from immune regulation, some recent works also demonstrated the influences of adipocyte-derived EVs on metabolic functions of macrophages. Flaherty III et al. provided evidences for the release of lipid-filled exosomes from mice adipocytes. These exosomes were taken up by bone marrow precursors *in vitro* and promoted their differentiation into ATM-like cells. This study revealed an alternative pathway for lipid metabolism, by which adipocyte-derived exosomes mediated the transportation of triglyceride into macrophages and subsequent hydrolysis, thus establishing a local lipid cycle to maintain homeostasis in perigonadal WAT (83) (**Table 1**). Indeed, besides triglyceride, fatty acids were also present in specific fraction of EVs from both human and mouse adipocytes, some of them were increased by obesity and influenced the functions of target cells through regulating metabolism (96, 97). With respect to the functions of these fatty acid-loaded EVs on macrophages, it remains an open but interesting area for future study. In addition, adipocyte-derived EVs also produce influence on the cholesterol metabolism of macrophages. Visceral fat from HFD-fed mice could release exosomes to induce the formation of macrophage foam cells by impairing their cholesterol efflux, thereby exacerbating atherosclerosis in hyperlipidemic apolipoprotein E-deficient mice (98). The similar effects on cholesterol efflux were also observed in EVs shed by human visceral fat, and the majority of these EVs were verified to be adipocyte origin (99). However, if the effects of EV miRNA specified in this study could be further verified, particularly for EVs from primary adipocytes, the above conclusion would be more convincing. Based on the roles of EVs, some treatments targeting the crosstalk between adipocytes and macrophages may provide protection against obesity-associated metabolic disorders by altering the release and regulatory effects of EVs from adipocytes. For instance, exosomes from melatonin-treated adipocytes inactivated Stat3/NF-κB signaling and alleviated HFD‐induced adipose inflammation, hepatic ER stress and steatosis *in vivo* (100, 101).

#### Action of Macrophage-Derived EVs on Adipocytes

In addition to taking up EVs as recipient cells, macrophages also release EVs to influence other cells. Earlier studies demonstrated that human THP-1 monocytes or differentiated macrophages produced EVs with specific miRNA, which could act on target cells to influence their functions, such as inducing monocyte differentiation into macrophages (102–106). Recently, several *in vitro* studies provided evidences for EV-mediated action on adipocytes by macrophages. Microvesicles from THP-1-differentiated M1 macrophages, which were induced by LPS plus IFN-γ, significantly induced insulin resistance in human adipocytes (107). While exosomes from THP-1-differentiated macrophages, which were induced by LPS, had no influences on insulin-mediated glucose uptake in human adipocytes, but changed their gene expression related to inflammation pathways (108). Moreover, high glucose promoted the package of miR-210 into exosomes from macrophages, which impaired glucose uptake and mitochondrial activity in 3T3-L1 adipocytes (109). These studies showed a slight difference in the effects of macrophage-derived EVs, possibly resulting from different stimuli on the macrophages, or different EV types carrying distinct components from the macrophages. Of note, these findings reflect the fact that both inflammatory and metabolic stresses could be passed from macrophages into adipocytes through EVs (**Table 1**).

Direct evidences for the regulation of ATMs on adipocyte functions come from the animal studies. Ying and colleagues isolated exosomes from mouse ATMs and observed their transportation into 3T3-L1 adipocytes through *in vitro* experiments. Interestingly, exosomes from either obese or lean ATMs produced different effects on insulin action in both insulin-target cells and animal models. Lean mice treated with obese ATM exosomes showed obvious insulin resistance, whereas obese mice treated with lean ATM exosomes showed improvement of insulin sensitivity. Consistently, exosomes from obese ATMs impaired insulin-induced AKT activation and glucose uptake in 3T3-L1 adipocytes, whereas exosomes from lean ATMs improved this process. Compared with lean ATM exosomes, obese ATM exosomes contained abundant miR-155, which might contribute to these undesired effects probably through targeting peroxisome proliferator-activated receptor (PPAR) -γ (110). In support of this study, another report also showed the impairment of insulin action in both 3T3-L1 adipocytes and lean mice by obese ATM exosomes, in which miR-29a was identified to be increased and exerted deleterious effects dependent on PPAR-δ (111). Meanwhile, both of the studies provided evidences for the effects of ATM exosomes on insulin sensitivity in hepatocytes and myocytes besides adipocytes (110, 111). The differences in exosomal miRNA profiles and their target pathways between these two studies remain to be clarified. Possible explanation might be the influence of differences in animal models, cell or exosome isolation methods and so on. Furthermore, another interesting study found a distinct population of lipid-laden ATMs with the capacity for exosome production, which could induce proinflammatory gene signature in adipose tissue similar to obese WAT, suggesting that ATMs could perform functions in both metabolism and immunity through EVs (60). Altogether, these findings have demonstrated the considerable impacts of ATM-derived EVs on local or systemic immunometabolism by targeting adipocytes as well as other cell types *via* paracrine and endocrine (**Table 1**).

### Adipose EVs in ADSC-Macrophage Interaction

In response to metabolic demands or stresses, ADSCs serve as the source of adipocytes that regulate glycolipid metabolism in adipose tissue. Meanwhile, as the main stromal cells, ADSCs also act as indispensable immune regulators inside or outside adipose tissue. During these processes, ADSCs and macrophages cooperate to form dynamic microenvironments to regulate immunometabolic homeostasis or disorders. Accumulating data have shown that either human or mouse ADSCs have an ability to promote the alternative activation of macrophages and inhibit the inflammation of monocytes/macrophages. These effects elicited by ADSCs provided protection against several inflammatory diseases in animal models such as experimental colitis, sepsis, hepatitis and neuroinflammation (43–46, 112–114). The early evidence for the regulation of ADSCs on monocytes or macrophages came from Gonzalez-Rey’s study, in which human ADSCs inhibited the production of TNF-α and IL-12 from activated macrophages of septic mice. More excitingly, systemic infusion of ADSCs protected against severe colitis and sepsis in animal models, suggesting the anti-inflammatory effects of ADSCs (44). Later, we demonstrated the strong ability of mouse ADSCs to promote the expression of IL-10 and arginase 1 in macrophages and their potential to inhibit obesity-induced WAT inflammation, further confirming the regulation of ADSCs on macrophages as well as inflammatory diseases (47). In a similar manner, ADSCs induced the alternative activation of macrophages and ameliorated colitis or neuroinflammation in animal models through several different pathways or inhibitory molecules like TSG-6 (43, 46, 112–114). Consistent with the roles of mouse ADSCs in obesity-induced WAT inflammation, human or rat ADSCs also displayed beneficial effects on relieving obesity-induced metabolic disorders, type 2 diabetes mellitus related complications in lung, liver, kidney, as well as cardiovascular diseases such as cardiac hypertrophy and aortic inflammation. And importantly, ADSC-induced macrophage polarization was actively involved in the above processes (47–50, 115–117). Conversely, macrophages with distinct phenotypes could also produce different effects on proliferation, differentiation and adipogenesis of ADSCs, either maintaining or disrupting local or systemic metabolic homeostasis (118–121). As both ADSCs and macrophages change their characteristics and functions with the alteration of metabolic status, instant information exchanges are necessary for the interaction between ADSCs and macrophages, in which soluble factors including EVs and cytokines are actively involved.

Regarding the communication of ADSCs with macrophages, several studies have provided evidences for the pivotal roles of soluble factors during these processes (122–124). Our previous study showed that ADSCs from lean mice attenuated obesity-induced WAT inflammation and metabolic disorders, in which soluble factors from ADSCs played critical roles in remodeling IL-10^high^ and arginase 1^high^ M2-like macrophages (47). Considering that ADSCs produce abundant EVs, it is inevitable to add EVs to the list of ADSC-macrophage dialogue. As expected, the delivery of exosomes from lean ADSCs into obese mice produced desirable effects on relieving obesity and improving insulin sensitivity in these mice. Exosomes from lean ADSCs promoted macrophage polarization toward arginase-1^high^ M2 phenotypes in visceral adipose tissue, which facilitated WAT beiging and homeostasis in HFD-fed mice. Using primary macrophages, we demonstrated that these exosomes could carry active Stat3 to promote M2 polarization and inhibit macrophage inflammation (125) (**Table 1**). Subsequently, several other studies confirmed that exosomes from either human or mouse ADSCs showed similar effects on macrophages, and more importantly, these exosomes were applied into the treatment of different disease models related inflammation or injury in animals (73, 126–130). Notably, the therapeutic potentials of ADSC-derived EVs have also been reported in metabolism-related disease models such as type 1, type 2 diabetes or diabetic nephropathy, and more target cells like T cells, hepatocytes or podocytes have been revealed (131–135). All these observations suggest that ADSC-derived EVs may regulate immunometabolic homeostasis in multiple ways. With respect to different types, contents of EVs from ADSCs as well as their detailed regulatory mechanisms, further investigation is still required. On the other hand, there are few reports regarding the action of ATM EVs on ADSCs, though some data showed that EVs from human monocytes influenced the expression of genes associated with cytokines or chemokines in human ADSCs (136).

## Conclusions and Perspectives

**Figure 2:**
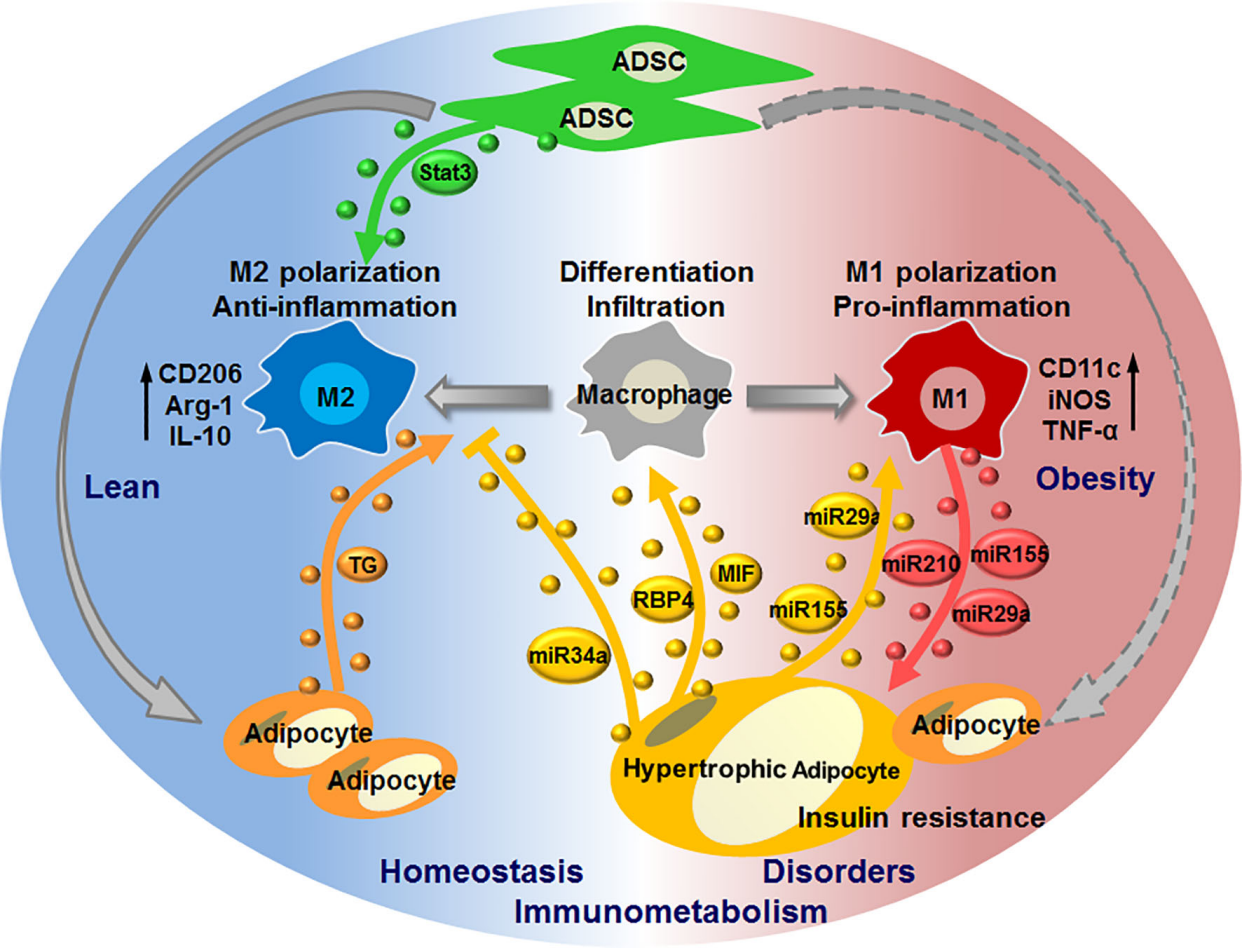
EV-mediated interaction of macrophages with adipocytes or ADSCs in regulating immunometabolic homeostasis or disorders. Under healthy condition, ADSCs release EVs carrying active Stat3 to induce macrophage polarization toward M2 phenotypes; adipocytes release EVs to transfer lipid (triglyceride, TG) into macrophages, thereby maintaining immunometabolic homeostasis in WAT. In case of metabolic stress like obesity, hypertrophic adipocytes secret EVs with MIF, RBP4, miR-155, miR-34a, miR-29a to promote macrophage infiltration and M1 polarization, whilst suppress M2 polarization; M1 macrophages in turn cause insulin resistance of adipocytes through delivering miR-155, miR-29a, miR-210 *via* EVs, thus forming a vicious cycle to promote immunometabolic disorders in WAT.

Recent studies provide evidences for the pivotal roles of adipose EVs that are produced by or act on macrophages in mediating adipocyte-macrophage dialogue and ADSC-macrophage interaction, as well as their roles in regulating local or systemic immunometabolism. Under physiological conditions, EVs released from ADSCs may dominate their interaction with macrophages to induce M2 phenotypes (IL-10^high^, arginase 1^high^), which facilitate the maintenance of immunometabolic homeostasis in WAT. While under pathological conditions such as obesity, EVs released from adipocytes, particularly hypertrophic adipocytes, may dominate their dialogue with macrophages to induce the infiltration and M1 polarization (TNF-α^high^, iNOS^high^) of macrophages. Macrophages switch their phenotypes to promote metabolic inflammation in WAT, and in turn release EVs to impair adipocyte functions, eventually forming a vicious cycle to aggravate immunometabolic disorders in WAT. Furthermore, adipocytes can also transfer lipid into ATMs by releasing EVs, which may mediate a lipid cycle to participate in immunometabolism (**Figure 2**).

Considering the complexity and diversity of adipose EVs, EVs from and to macrophages in immunometabolism remain to be further elucidated. For future studies in this field, several directions may be helpful. From the spatial dimension, various of EVs with distinct cell sources and targets need to be determined for their special contents and functions in specific microenvironments. From the time dimension, instant alterations of EV types, numbers and contents are required to be monitored with the change of immune or metabolic microenvironments. From the perspective of application, more promising EV-based biomarkers and therapeutic approaches are still awaiting to be identified and constructed.

## Author Contributions

ZZ and YT drafted the manuscript. HZ revised and edited the manuscript. ZZ designed and created the figures and tables. QW wrote and revised the manuscript, designed the figures and tables. All authors contributed to the article and approved the submitted version.

## Funding

This work is supported by the National Natural Science Foundation of China (81770838, 81970733, 81471065).

## Conflict of Interest

The authors declare that the research was conducted in the absence of any commercial or financial relationships that could be construed as a potential conflict of interest.

## References

[1] Chouchani ET Kajimura S. Metabolic Adaptation and Maladaptation in Adipose Tissue. Nat Metab (2019) 1(2):189–200. 10.1038/s42255-018-0021-8 31903450 PMC6941795

[2] Trayhurn P Beattie JH. Physiological Role of Adipose Tissue: White Adipose Tissue as an Endocrine and Secretory Organ. Proc Nutr Soc (2001) 60(3):329–39. 10.1079/PNS200194 11681807

[3] Cannon B Nedergaard J. Brown Adipose Tissue: Function and Physiological Significance. Physiol Rev (2004) 84(1):277–359. 10.1152/physrev.00015.2003 14715917

[4] Brestoff JR Artis D. Immune Regulation of Metabolic Homeostasis in Health and Disease. Cell (2015) 161(1):146–60. 10.1016/j.cell.2015.02.022 PMC4400287 25815992

[5] Scheja L Heeren J. The Endocrine Function of Adipose Tissues in Health and Cardiometabolic Disease. Nat Rev Endocrinol (2019) 15(9):507–24. 10.1038/s41574-019-0230-6 31296970

[6] Wang Q Wu H. T Cells in Adipose Tissue: Critical Players in Immunometabolism. Front Immunol (2018) 9:2509. 10.3389/fimmu.2018.02509 30459770 PMC6232870

[7] Gregor MF Hotamisligil GS. Inflammatory Mechanisms in Obesity. Annu Rev Immunol (2011) 29:415–45. 10.1146/annurev-immunol-031210-101322 21219177

[8] Hotamisligil GS. Inflammation, Metaflammation and Immunometabolic Disorders. Nature (2017) 542(7640):177–85. 10.1038/nature21363 28179656

[9] Hotamisligil GS. Foundations of Immunometabolism and Implications for Metabolic Health and Disease. Immunity (2017) 47(3):406–20. 10.1016/j.immuni.2017.08.009 PMC5627521 28930657

[10] Man K Kutyavin VI Chawla A. Tissue Immunometabolism: Development, Physiology, and Pathobiology. Cell Metab (2017) 25(1):11–26. 10.1016/j.cmet.2016.08.016 27693378 PMC5226870

[11] Stern JH Rutkowski JM Scherer PE. Adiponectin, Leptin, and Fatty Acids in the Maintenance of Metabolic Homeostasis Through Adipose Tissue Crosstalk. Cell Metab (2016) 23(5):770–84. 10.1016/j.cmet.2016.04.011 PMC4864949 27166942

[12] Glass CK Olefsky JM. Inflammation and Lipid Signaling in the Etiology of Insulin Resistance. Cell Metab (2012) 15(5):635–45. 10.1016/j.cmet.2012.04.001 PMC4156155 22560216

[13] Antonyak MA Cerione RA. Emerging Picture of the Distinct Traits and Functions of Microvesicles and Exosomes. Proc Natl Acad Sci (2015) 112(12):3589–90. 10.1073/pnas.1502590112 PMC4378426 25762069

[14] Kanada M Bachmann MH Hardy JW Frimannson DO Bronsart L Wang A. Differential Fates of Biomolecules Delivered to Target Cells Via Extracellular Vesicles. Proc Natl Acad Sci (2015) 112(12):E1433–42. 10.1073/pnas.1418401112 PMC4378439 25713383

[15] Margolis L Sadovsky Y. The Biology of Extracellular Vesicles: The Known Unknowns. PLoS Biol (2019) 17(7):e3000363. 10.1371/journal.pbio.3000363 31318874 PMC6667152

[16] Stahl PD Raposo G. Extracellular Vesicles: Exosomes and Microvesicles, Integrators of Homeostasis. Physiol (Bethesda) (2019) 34(3):169–77. 10.1152/physiol.00045.2018 30968753

[17] Huang-Doran I Zhang CY Vidal-Puig A. Extracellular Vesicles: Novel Mediators of Cell Communication In Metabolic Disease. Trends Endocrinol Metab (2017) 28(1):3–18. 10.1016/j.tem.2016.10.003 27810172

[18] Rodbell M. Metabolism of Isolated Fat Cells. I. Effects of Hormones on Glucose Metabolism and Lipolysis. J Biol Chem (1964) 239:375–80. 10.1016/S0021-9258(18)51687-2 14169133

[19] Eto H Suga H Matsumoto D Inoue K Aoi N Kato H. Characterization of Structure and Cellular Components of Aspirated and Excised Adipose Tissue. Plast Reconstruct Surg (2009) 124(4):1087–97. 10.1097/PRS.0b013e3181b5a3f1 19935292

[20] Brown JC Shang H Li Y Yang N Patel N Katz AJ. Isolation of Adipose-Derived Stromal Vascular Fraction Cells Using a Novel Point-of-Care Device: Cell Characterization and Review of the Literature. Tissue Eng Part C: Methods (2017) 23(3):125–35. 10.1089/ten.tec.2016.0377 28177263

[21] Zuk PA Zhu M Mizuno H Huang J Futrell JW Katz AJ. Multilineage Cells From Human Adipose Tissue: Implications for Cell-Based Therapies. Tissue Eng (2001) 7(2):211–28. 10.1089/107632701300062859 11304456

[22] Wu H Ballantyne CM. Metabolic Inflammation and Insulin Resistance in Obesity. Circ Res (2020) 126(11):1549–64. 10.1161/CIRCRESAHA.119.315896 PMC7250139 32437299

[23] McLaughlin T Ackerman SE Shen L Engleman E. Role of Innate and Adaptive Immunity in Obesity-Associated Metabolic Disease. J Clin Invest (2017) 127(1):5–13. 10.1172/JCI88876 28045397 PMC5199693

[24] Rochette L Mazini L Malka G Zeller M Cottin Y Vergely C. The Crosstalk of Adipose-Derived Stem Cells (ADSC), Oxidative Stress, and Inflammation in Protective and Adaptive Responses. Int J Mol Sci (2020) 21(23):9262. 10.3390/ijms21239262 PMC7730805 33291664

[25] Song J Deng T. The Adipocyte and Adaptive Immunity. Front Immunol (2020) 11:593058. 10.3389/fimmu.2020.593058 33329579 PMC7728694

[26] Hui X Zhang M Gu P Li K Gao Y Wu D. Adipocyte SIRT1 Controls Systemic Insulin Sensitivity by Modulating Macrophages in Adipose Tissue. EMBO Rep (2017) 18(4):645–57. 10.15252/embr.201643184 PMC5376747 28270525

[27] Hotamisligil GS Shargill NS Spiegelman BM. Adipose Expression of Tumor Necrosis Factor-Alpha: Direct Role in Obesity-Linked Insulin Resistance. Science (1993) 259(5091):87–91. 10.1126/science.7678183 7678183

[28] Lefterova MI Lazar MA. New Developments in Adipogenesis. Trends Endocrinol Metab (2009) 20(3):107–14. 10.1016/j.tem.2008.11.005 19269847

[29] Steppan CM Bailey ST Bhat S Brown EJ Banerjee RR Wright CM. The Hormone Resistin Links Obesity to Diabetes. Nature (2001) 409(6818):307–12. 10.1038/35053000 11201732

[30] Shoelson SE Lee J Goldfine AB. Inflammation and Insulin Resistance. J Clin Invest (2006) 116(7):1793–801. 10.1172/JCI29069 PMC1483173 16823477

[31] Fedorenko A Lishko PV Kirichok Y. Mechanism of Fatty-Acid-Dependent UCP1 Uncoupling in Brown Fat Mitochondria. Cell (2012) 151(2):400–13. 10.1016/j.cell.2012.09.010 PMC3782081 23063128

[32] Nedergaard J Bengtsson T Cannon B. New Powers of Brown Fat: Fighting the Metabolic Syndrome. Cell Metab (2011) 13(3):238–40. 10.1016/j.cmet.2011.02.009 21356513

[33] Shinoda K Luijten IHN Hasegawa Y Hong H Sonne SB Kim M. Genetic and Functional Characterization of Clonally Derived Adult Human Brown Adipocytes. Nat Med (2015) 21(4):389–94. 10.1038/nm.3819 PMC4427356 25774848

[34] Sidossis L Kajimura S. Brown and Beige Fat in Humans: Thermogenic Adipocytes That Control Energy and Glucose Homeostasis. J Clin Invest (2015) 125(2):478–86. 10.1172/JCI78362 PMC4319444 25642708

[35] Harms M Seale P. Brown and Beige Fat: Development, Function and Therapeutic Potential. Nat Med (2013) 19(10):1252–63. 10.1038/nm.3361 24100998

[36] Hu X Cifarelli V Sun S Kuda O Abumrad NA Su X. Major Role of Adipocyte Prostaglandin E2 in Lipolysis-Induced Macrophage Recruitment. J Lipid Res (2016) 57(4):663–73. 10.1194/jlr.M066530 PMC4808775 26912395

[37] Suganami T Tanimoto-Koyama K Nishida J Itoh M Yuan X Mizuarai S. Role of the Toll-like Receptor 4/Nf-κb Pathway in Saturated Fatty Acid–Induced Inflammatory Changes in the Interaction Between Adipocytes and Macrophages. Arterioscler Thromb Vasc Biol (2007) 27(1):84–91. 10.1161/01.ATV.0000251608.09329.9a 17082484

[38] Furuhashi M Fucho R Gorgun CZ Tuncman G Cao H Hotamisligil GS. Adipocyte/Macrophage Fatty Acid-Binding Proteins Contribute to Metabolic Deterioration Through Actions in Both Macrophages and Adipocytes in Mice. J Clin Invest (2008) 118(7):2640–50. 10.1172/JCI34750 PMC2423863 18551191

[39] Deng T Lyon CJ Minze LJ Lin J Zou J Liu JZ. Class II Major Histocompatibility Complex Plays an Essential Role in Obesity-Induced Adipose Inflammation. Cell Metab (2013) 17(3):411–22. 10.1016/j.cmet.2013.02.009 PMC3619392 23473035

[40] Huh JY Kim JI Park YJ Hwang IJ Lee YS Sohn JH. A Novel Function of Adipocytes in Lipid Antigen Presentation to iNKT Cells. Mol Cell Biol (2013) 33(2):328–39. 10.1128/MCB.00552-12 PMC3554106 23149942

[41] Stafeev I Podkuychenko N Michurina S Sklyanik I Panevina A Shestakova E. Low Proliferative Potential of Adipose-Derived Stromal Cells Associates With Hypertrophy and Inflammation in Subcutaneous and Omental Adipose Tissue of Patients With Type 2 Diabetes Mellitus. J Diabetes Complicat (2019) 33(2):148–59. 10.1016/j.jdiacomp.2018.10.011 30482492

[42] Pan Z Zhou Z Zhang H Zhao H Song P Wang D. CD90 Serves as Differential Modulator of Subcutaneous and Visceral Adipose-Derived Stem Cells by Regulating AKT Activation That Influences Adipose Tissue and Metabolic Homeostasis. Stem Cell Res Ther (2019) 10(1):355. 10.1186/s13287-019-1459-7 31779686 PMC6883612

[43] Bowles AC Wise RM Gerstein BY Thomas RC Ogelman R Febbo I. Immunomodulatory Effects of Adipose Stromal Vascular Fraction Cells Promote Alternative Activation Macrophages to Repair Tissue Damage. Stem Cells (2017) 35(10):2198–207. 10.1002/stem.2689 28801931

[44] Gonzalez-Rey E Anderson P Gonzalez MA Rico L Buscher D Delgado M. Human Adult Stem Cells Derived From Adipose Tissue Protect Against Experimental Colitis and Sepsis. Gut (2009) 58(7):929–39. 10.1136/gut.2008.168534 19136511

[45] Higashimoto M Sakai Y Takamura M Usui S Nasti A Yoshida K. Adipose Tissue Derived Stromal Stem Cell Therapy in Murine ConA-derived Hepatitis Is Dependent on Myeloid-Lineage and CD4+ T-Cell Suppression. Eur J Immunol (2013) 43(11):2956–68. 10.1002/eji.201343531 23934743

[46] Kawata Y Tsuchiya A Seino S Watanabe Y Kojima Y Ikarashi S. Early Injection of Human Adipose Tissue-Derived Mesenchymal Stem Cell After Inflammation Ameliorates Dextran Sulfate Sodium-Induced Colitis in Mice Through the Induction of M2 Macrophages and Regulatory T Cells. Cell Tissue Res (2019) 376(2):257–71. 10.1007/s00441-018-02981-w 30635774

[47] Shang Q Bai Y Wang G Song Q Guo C Zhang L. Delivery of Adipose-Derived Stem Cells Attenuates Adipose Tissue Inflammation and Insulin Resistance in Obese Mice Through Remodeling Macrophage Phenotypes. Stem Cells Dev (2015) 24(17):2052–64. 10.1089/scd.2014.0557 25923535

[48] Shree N Venkategowda S Venkatranganna MV Datta I Bhonde RR. Human Adipose Tissue Mesenchymal Stem Cells as a Novel Treatment Modality for Correcting Obesity Induced Metabolic Dysregulation. Int J Obes (Lond) (2019) 43(10):2107–18. 10.1038/s41366-019-0438-5 31462691

[49] Hwang I Jo K Shin KC Kim JI Ji Y Park YJ. GABA-stimulated adipose-derived stem cells suppress subcutaneous adipose inflammation in obesity. Proc Natl Acad Sci USA (2019) 116(24):11936–45. 10.1073/pnas.1822067116 PMC6575165 31160440

[50] Yu S Cheng Y Zhang L Yin Y Xue J Li B. Treatment With Adipose Tissue-Derived Mesenchymal Stem Cells Exerts Anti-Diabetic Effects, Improves Long-Term Complications, and Attenuates Inflammation in Type 2 Diabetic Rats. Stem Cell Res Ther (2019) 10(1):333. 10.1186/s13287-019-1474-8 31747961 PMC6868748

[51] Weisberg SP McCann D Desai M Rosenbaum M Leibel RL Ferrante AW. Obesity Is Associated With Macrophage Accumulation in Adipose Tissue. J Clin Invest (2003) 112(12):1796–808. 10.1172/JCI200319246 PMC296995 14679176

[52] Xu H Barnes GT Yang Q Tan G Yang D Chou CJ. Chronic Inflammation in Fat Plays A Crucial Role in the Development of Obesity-Related Insulin Resistance. J Clin Invest (2003) 112(12):1821–30. 10.1172/JCI200319451 PMC296998 14679177

[53] Serbina NV Pamer EG. Monocyte Emigration From Bone Marrow During Bacterial Infection Requires Signals Mediated by Chemokine Receptor CCR2. Nat Immunol (2006) 7(3):311–7. 10.1038/ni1309 16462739

[54] Cinti S Mitchell G Barbatelli G Murano I Ceresi E Faloia E. Adipocyte Death Defines Macrophage Localization and Function in Adipose Tissue of Obese Mice and Humans. J Lipid Res (2005) 46(11):2347–55. 10.1194/jlr.M500294-JLR200 16150820

[55] Lumeng CN Bodzin JL Saltiel AR. Obesity Induces a Phenotypic Switch in Adipose Tissue Macrophage Polarization. J Clin Invest (2007) 117(1):175–84. 10.1172/JCI29881 PMC1716210 17200717

[56] Gordon S Taylor PR. Monocyte and Macrophage Heterogeneity. Nat Rev Immunol (2005) 5(12):953–64. 10.1038/nri1733 16322748

[57] Fujisaka S Usui I Bukhari A Ikutani M Oya T Kanatani Y. Regulatory Mechanisms for Adipose Tissue M1 and M2 Macrophages in Diet-Induced Obese Mice. Diabetes (2009) 58(11):2574–82. 10.2337/db08-1475 PMC2768159 19690061

[58] Krogh-Madsen R Plomgaard P Keller P Keller C Pedersen BK. Insulin Stimulates Interleukin-6 and Tumor Necrosis Factor-Alpha Gene Expression in Human Subcutaneous Adipose Tissue. Am J Physiol Endocrinol Metab (2004) 286(2):E234–8. 10.1152/ajpendo.00274.2003 14532168

[59] Gao D Madi M Ding C Fok M Steele T Ford C. Interleukin-1beta Mediates Macrophage-Induced Impairment of Insulin Signaling in Human Primary Adipocytes. Am J Physiol Endocrinol Metab (2014) 307(3):E289–304. 10.1152/ajpendo.00430.2013 PMC4121578 24918199

[60] Hill DA Lim HW Kim YH Ho WY Foong YH Nelson VL. Distinct macrophage populations direct inflammatory versus physiological changes in adipose tissue. Proc Natl Acad Sci USA (2018) 115(22):E5096–105. 10.1073/pnas.1802611115 PMC5984532 29760084

[61] Xu R Greening DW Zhu HJ Takahashi N Simpson RJ. Extracellular Vesicle Isolation and Characterization: Toward Clinical Application. J Clin Invest (2016) 126(4):1152–62. 10.1172/JCI81129 PMC4811150 27035807

[62] Gyorgy B Szabo TG Pasztoi M Pal Z Misjak P Aradi B. Membrane Vesicles, Current State-of-the-Art: Emerging Role of Extracellular Vesicles. Cell Mol Life Sci (2011) 68(16):2667–88. 10.1007/s00018-011-0689-3 PMC3142546 21560073

[63] Kalluri R LeBleu VS. The Biology, Function, and Biomedical Applications of Exosomes. Science (2020) 367(6478):eaau6977. 10.1126/science.aau6977 32029601 PMC7717626

[64] Kalra H Drummen GP Mathivanan S. Focus on Extracellular Vesicles: Introducing the Next Small Big Thing. Int J Mol Sci (2016) 17(2):170. 10.3390/ijms17020170 26861301 PMC4783904

[65] Raposo G Stoorvogel W. Extracellular Vesicles: Exosomes, Microvesicles, and Friends. J Cell Biol (2013) 200(4):373–83. 10.1083/jcb.201211138 PMC3575529 23420871

[66] Mathieu M Martin-Jaular L Lavieu G Thery C. Specificities of Secretion and Uptake of Exosomes and Other Extracellular Vesicles for Cell-to-Cell Communication. Nat Cell Biol (2019) 21(1):9–17. 10.1038/s41556-018-0250-9 30602770

[67] Mulcahy LA Pink RC Carter DR. Routes and Mechanisms of Extracellular Vesicle Uptake. J Extracell Vesicles (2014) 3:24641. 10.3402/jev.v3.24641 PMC4122821 25143819

[68] Obata Y Kita S Koyama Y Fukuda S Takeda H Takahashi M. Adiponectin/T-Cadherin System Enhances Exosome Biogenesis and Decreases Cellular Ceramides by Exosomal Release. JCI Insight (2018) 3(8):e99680. 10.1172/jci.insight.99680 PMC5931116 29669945

[69] Kralisch S Ebert T Lossner U Jessnitzer B Stumvoll M Fasshauer M. Adipocyte Fatty Acid-Binding Protein Is Released From Adipocytes by a Non-Conventional Mechanism. Int J Obes (Lond) (2014) 38(9):1251–4. 10.1038/ijo.2013.232 24445660

[70] Kranendonk ME Visseren FL van Balkom BW Nolte-’t Hoen EN van Herwaarden JA de Jager W. Human Adipocyte Extracellular Vesicles in Reciprocal Signaling Between Adipocytes and Macrophages. Obes (Silver Spring) (2014) 22(5):1296–308. 10.1002/oby.20679 24339422

[71] Eguchi A Lazic M Armando AM Phillips SA Katebian R Maraka S. Circulating Adipocyte-Derived Extracellular Vesicles Are Novel Markers of Metabolic Stress. J Mol Med (2016) 94(11):1241–53. 10.1007/s00109-016-1446-8 PMC5071132 27394413

[72] Crewe C Joffin N Rutkowski JM Kim M Zhang F Towler DA. An Endothelial-to-Adipocyte Extracellular Vesicle Axis Governed by Metabolic State. Cell (2018) 175(3):695–708.e13. 10.1016/j.cell.2018.09.005 30293865 PMC6195477

[73] Lo Sicco C Reverberi D Balbi C Ulivi V Principi E Pascucci L. Mesenchymal Stem Cell-Derived Extracellular Vesicles as Mediators of Anti-Inflammatory Effects: Endorsement of Macrophage Polarization. Stem Cells Trans Med (2017) 6(3):1018–28. 10.1002/sctm.16-0363 PMC5442783 28186708

[74] Kranendonk ME Visseren FL van Herwaarden JA Nolte-’t Hoen EN de Jager W Wauben MH. Effect of Extracellular Vesicles of Human Adipose Tissue on Insulin Signaling in Liver and Muscle Cells. Obes (Silver Spring) (2014) 22(10):2216–23. 10.1002/oby.20847 25045057

[75] Koeck ES Iordanskaia T Sevilla S Ferrante SC Hubal MJ Freishtat RJ. Adipocyte Exosomes Induce Transforming Growth Factor Beta Pathway Dysregulation in Hepatocytes: A Novel Paradigm for Obesity-Related Liver Disease. J Surg Res (2014) 192(2):268–75. 10.1016/j.jss.2014.06.050 25086727

[76] Santamaria-Martos F Benitez ID Latorre J Lluch A Moreno-Navarrete JM Sabater M. Comparative and Functional Analysis of Plasma Membrane-Derived Extracellular Vesicles From Obese vs. Nonobese Women. Clin Nutr (2020) 39(4):1067–76. 10.1016/j.clnu.2019.04.008 31036413

[77] Ferrante SC Nadler EP Pillai DK Hubal MJ Wang Z Wang JM. Adipocyte-Derived Exosomal miRNAs: A Novel Mechanism for Obesity-Related Disease. Pediatr Res (2014) 77(3):447–54. 10.1038/pr.2014.202 PMC4346410 25518011

[78] Zhang HG Liu C Su K Yu S Zhang L Zhang S. A Membrane Form of TNF-alpha Presented by Exosomes Delays T Cell Activation-Induced Cell Death. J Immunol (2006) 176(12):7385–93. 10.4049/jimmunol.176.12.7385 16751383

[79] Fitzgerald W Freeman ML Lederman MM Vasilieva E Romero R Margolis L. A System of Cytokines Encapsulated in ExtraCellular Vesicles. Sci Rep (2018) 8(1):8973. 10.1038/s41598-018-27190-x 29895824 PMC5997670

[80] Barnes BJ Somerville CC. Modulating Cytokine Production Via Select Packaging and Secretion From Extracellular Vesicles. Front Immunol (2020) 11:1040. 10.3389/fimmu.2020.01040 32547552 PMC7272603

[81] Aiello A Giannessi F Percario ZA Affabris E. An Emerging Interplay Between Extracellular Vesicles and Cytokines. Cytokine Growth Factor Rev (2020) 51:49–60. 10.1016/j.cytogfr.2019.12.003 31874738

[82] Jaitin DA Adlung L Thaiss CA Weiner A Li B Descamps H. Lipid-Associated Macrophages Control Metabolic Homeostasis in a Trem2-Dependent Manner. Cell (2019) 178(3):686–98.e14. 10.1016/j.cell.2019.05.054 31257031 PMC7068689

[83] Flaherty SE 3rd Grijalva A Xu X Ables E Nomani A Ferrante AW Jr. A Lipase-Independent Pathway of Lipid Release and Immune Modulation by Adipocytes. Science (2019) 363(6430):989–93. 10.1126/science.aaw2586 PMC6579605 30819964

[84] Hubal MJ Nadler EP Ferrante SC Barberio MD Suh JH Wang J. Circulating Adipocyte-Derived Exosomal MicroRNAs Associated With Decreased Insulin Resistance After Gastric Bypass. Obes (Silver Spring) (2017) 25(1):102–10. 10.1002/oby.21709 PMC5182153 27883272

[85] Thomou T Mori MA Dreyfuss JM Konishi M Sakaguchi M Wolfrum C. Adipose-Derived Circulating miRNAs Regulate Gene Expression in Other Tissues. Nature (2017) 542(7642):450–5. 10.1038/nature21365 PMC5330251 28199304

[86] Connolly KD Wadey RM Mathew D Johnson E Rees DA James PE. Evidence for Adipocyte-Derived Extracellular Vesicles in the Human Circulation. Endocrinology (2018) 159(9):3259–67. 10.1210/en.2018-00266 PMC6109300 30016424

[87] Mleczko J Ortega FJ Falcon-Perez JM Wabitsch M Fernandez-Real JM Mora S. Extracellular Vesicles From Hypoxic Adipocytes and Obese Subjects Reduce Insulin-Stimulated Glucose Uptake. Mol Nutr Food Res (2018) 62(5):1700917. 10.1002/mnfr.201700917 PMC5887919 29292863

[88] Gao J Li X Wang Y Cao Y Yao D Sun L. Adipocyte-Derived Extracellular Vesicles Modulate Appetite and Weight Through mTOR Signalling in the Hypothalamus. Acta Physiol (Oxf) (2020) 228(2):e13339. 10.1111/apha.13339 31278836

[89] Song Y Li H Ren X Li H Feng C. SNHG9, Delivered by Adipocyte-Derived Exosomes, Alleviates Inflammation and Apoptosis of Endothelial Cells Through Suppressing TRADD Expression. Eur J Pharmacol (2020) 872:172977. 10.1016/j.ejphar.2020.172977 32007500

[90] Deng ZB Poliakov A Hardy RW Clements R Liu C Liu Y. Adipose Tissue Exosome-Like Vesicles Mediate Activation of Macrophage-Induced Insulin Resistance. Diabetes (2009) 58(11):2498–505. 10.2337/db09-0216 PMC2768161 19675137

[91] Renovato-Martins M Matheus ME de Andrade IR Moraes JA da Silva SV Citelli dos Reis M. Microparticles Derived From Obese Adipose Tissue Elicit a Pro-Inflammatory Phenotype of CD16 +, CCR5 + and TLR8 + Monocytes. Biochim Biophys Acta (BBA) - Mol Basis Dis (2017) 1863(1):139–51. 10.1016/j.bbadis.2016.09.016 27677832

[92] Eguchi A Mulya A Lazic M Radhakrishnan D Berk MP Povero D. Microparticles Release by Adipocytes Act as “Find-Me” Signals to Promote Macrophage Migration. PLoS One (2015) 10(4):e0123110. 10.1371/journal.pone.0123110 25849214 PMC4388837

[93] Tamara C Nerea LB Belen BS Aurelio S Ivan C Fernando S. Vesicles Shed by Pathological Murine Adipocytes Spread Pathology: Characterization and Functional Role of Insulin Resistant/Hypertrophied Adiposomes. Int J Mol Sci (2020) 21(6):2252. 10.3390/ijms21062252 PMC7139903 32214011

[94] Pan Y Hui X Hoo RLC Ye D Chan CYC Feng T. Adipocyte-Secreted Exosomal microRNA-34a Inhibits M2 Macrophage Polarization to Promote Obesity-Induced Adipose Inflammation. J Clin Invest (2019) 129(2):834–49. 10.1172/JCI123069 PMC6355214 30667374

[95] Zhang Y Mei H Chang X Chen F Zhu Y Han X. Adipocyte-Derived Microvesicles From Obese Mice Induce M1 Macrophage Phenotype Through Secreted Mir-155. J Mol Cell Biol (2016) 8(6):505–17. 10.1093/jmcb/mjw040 27671445

[96] Clement E Lazar I Attane C Carrie L Dauvillier S Ducoux-Petit M. Adipocyte Extracellular Vesicles Carry Enzymes and Fatty Acids That Stimulate Mitochondrial Metabolism and Remodeling in Tumor Cells. EMBO J (2020) 39(3):e102525. 10.15252/embj.2019102525 31919869 PMC6996584

[97] Connolly KD Guschina IA Yeung V Clayton A Draman MS Von Ruhland C. Characterisation of Adipocyte-Derived Extracellular Vesicles Released Pre- and Post-Adipogenesis. J Extracell Vesicles (2015) 4:29159. 10.3402/jev.v4.29159 26609807 PMC4661001

[98] Xie Z Wang X Liu X Du H Sun C Shao X. Adipose-Derived Exosomes Exert Proatherogenic Effects by Regulating Macrophage Foam Cell Formation and Polarization. J Am Heart Assoc (2018) 7(5):e007442. 10.1161/JAHA.117.007442 29502100 PMC5866320

[99] Barberio MD Kasselman LJ Playford MP Epstein SB Renna HA Goldberg M. Cholesterol Efflux Alterations in Adolescent Obesity: Role of Adipose-Derived Extracellular Vesical Micrornas. J Transl Med (2019) 17(1):232. 10.1186/s12967-019-1980-6 31331347 PMC6647309

[100] Liu Z Gan L Zhang T Ren Q Sun C. Melatonin Alleviates Adipose Inflammation Through Elevating Alpha-Ketoglutarate and Diverting Adipose-Derived Exosomes to Macrophages in Mice. J Pineal Res (2018) 64(1):e12455. 10.1111/jpi.12455 29149454

[101] Rong B Feng R Liu C Wu Q Sun C. Reduced Delivery of Epididymal Adipocyte-Derived Exosomal Resistin is Essential for Melatonin Ameliorating Hepatic Steatosis in Mice. J Pineal Res (2019) 66(4):e12561. 10.1111/jpi.12561 30659651

[102] Zhang Y Liu D Chen X Li J Li L Bian Z. Secreted Monocytic miR-150 Enhances Targeted Endothelial Cell Migration. Mol Cell (2010) 39(1):133–44. 10.1016/j.molcel.2010.06.010 20603081

[103] Ismail N Wang Y Dakhlallah D Moldovan L Agarwal K Batte K. Macrophage Microvesicles Induce Macrophage Differentiation and miR-223 Transfer. Blood (2013) 121(6):984–95. 10.1182/blood-2011-08-374793 PMC3567345 23144169

[104] McDonald MK Tian Y Qureshi RA Gormley M Ertel A Gao R. Functional Significance of Macrophage-Derived Exosomes in Inflammation and Pain. Pain (2014) 155(8):1527–39. 10.1016/j.pain.2014.04.029 PMC4106699 24792623

[105] Afonyushkin T Binder CJ. Extracellular Vesicles Act as Messengers of Macrophages Sensing Atherogenic Stimuli. Arterioscler Thromb Vasc Biol (2018) 38(1):2–3. 10.1161/ATVBAHA.117.310257 29282243

[106] Hulsmans M Holvoet P. MicroRNA-containing Microvesicles Regulating Inflammation in Association With Atherosclerotic Disease. Cardiovasc Res (2013) 100(1):7–18. 10.1093/cvr/cvt161 23774505

[107] Zhang Y Shi L Mei H Zhang J Zhu Y Han X. Inflamed Macrophage Microvesicles Induce Insulin Resistance in Human Adipocytes. Nutr Metab (Lond) (2015) 12:21. 10.1186/s12986-015-0016-3 26064180 PMC4462080

[108] De Silva N Samblas M Martinez JA Milagro FI. Effects of Exosomes From LPS-activated Macrophages on Adipocyte Gene Expression, Differentiation, and Insulin-Dependent Glucose Uptake. J Physiol Biochem (2018) 74(4):559–68. 10.1007/s13105-018-0622-4 29560554

[109] Tian F Tang P Sun Z Zhang R Zhu D He J. miR-210 in Exosomes Derived From Macrophages Under High Glucose Promotes Mouse Diabetic Obesity Pathogenesis by Suppressing Ndufa4 Expression. J Diabetes Res (2020) 2020:1–12. 10.1155/2020/6894684 PMC7106924 32258168

[110] Ying W Riopel M Bandyopadhyay G Dong Y Birmingham A Seo JB. Adipose Tissue Macrophage-Derived Exosomal Mirnas Can Modulate In Vivo and In Vitro Insulin Sensitivity. Cell (2017) 171(2):372–84.e12. 10.1016/j.cell.2017.08.035 28942920

[111] Liu T Sun YC Cheng P Shao HG. Adipose Tissue Macrophage-Derived Exosomal miR-29a Regulates Obesity-Associated Insulin Resistance. Biochem Biophys Res Commun (2019) 515(2):352–8. 10.1016/j.bbrc.2019.05.113 31153636

[112] Song WJ Li Q Ryu MO Ahn JO Bhang DH Jung YC. TSG-6 Released From Intraperitoneally Injected Canine Adipose Tissue-Derived Mesenchymal Stem Cells Ameliorate Inflammatory Bowel Disease by Inducing M2 Macrophage Switch in Mice. Stem Cell Res Ther (2018) 9(1):91. 10.1186/s13287-018-0841-1 29625582 PMC5889600

[113] Zhou Z Tian X Mo B Xu H Zhang L Huang L. Adipose Mesenchymal Stem Cell Transplantation Alleviates Spinal Cord Injury-Induced Neuroinflammation Partly by Suppressing the Jagged1/Notch Pathway. Stem Cell Res Ther (2020) 11(1):212. 10.1186/s13287-020-01724-5 32493480 PMC7268310

[114] Park HJ Kim J Saima FT Rhee KJ Hwang S Kim MY. Adipose-Derived Stem Cells Ameliorate Colitis by Suppression of Inflammasome Formation and Regulation of M1-macrophage Population Through Prostaglandin E2. Biochem Biophys Res Commun (2018) 498(4):988–95. 10.1016/j.bbrc.2018.03.096 29550474

[115] Lee TM Harn HJ Chiou TW Chuang MH Chen CH Chuang CH. Remote Transplantation of Human Adipose-Derived Stem Cells Induces Regression of Cardiac Hypertrophy by Regulating the Macrophage Polarization in Spontaneously Hypertensive Rats. Redox Biol (2019) 27:101170. 10.1016/j.redox.2019.101170 31164286 PMC6859583

[116] Liu J Qiu P Qin J Wu X Wang X Yang X. Allogeneic Adipose-Derived Stem Cells Promote Ischemic Muscle Repair by Inducing M2 Macrophage Polarization Via the HIF-1alpha/IL-10 Pathway. Stem Cells (2020) 38(10):1307–20. 10.1002/stem.3250 PMC7590195 32627897

[117] Xie J Jones TJ Feng D Cook TG Jester AA Yi R. Human Adipose-Derived Stem Cells Suppress Elastase-Induced Murine Abdominal Aortic Inflammation and Aneurysm Expansion Through Paracrine Factors. Cell Transplant (2017) 26(2):173–89. 10.3727/096368916X692212 PMC5657756 27436185

[118] Bilkovski R Schulte DM Oberhauser F Mauer J Hampel B Gutschow C. Adipose Tissue Macrophages Inhibit Adipogenesis of Mesenchymal Precursor Cells Via wnt-5a in Humans. Int J Obes (Lond) (2011) 35(11):1450–4. 10.1038/ijo.2011.6 21285942

[119] Cho YK Son Y Kim SN Song HD Kim M Park JH. MicroRNA-10a-5p Regulates Macrophage Polarization and Promotes Therapeutic Adipose Tissue Remodeling. Mol Metab (2019) 29:86–98. 10.1016/j.molmet.2019.08.015 31668395 PMC6734158

[120] Nawaz A Aminuddin A Kado T Takikawa A Yamamoto S Tsuneyama K. Cd206(+) M2-like Macrophages Regulate Systemic Glucose Metabolism by Inhibiting Proliferation of Adipocyte Progenitors. Nat Commun (2017) 8(1):286. 10.1038/s41467-017-00231-1 28819169 PMC5561263

[121] Ren X Fu X Zhang X Chen S Huang S Yao L. Testosterone Regulates 3T3-L1 Pre-Adipocyte Differentiation and Epididymal Fat Accumulation in Mice Through Modulating Macrophage Polarization. Biochem Pharmacol (2017) 140:73–88. 10.1016/j.bcp.2017.05.022 28642037

[122] Sun M Sun L Huang C Chen BC Zhou Z. Induction of Macrophage M2b/C Polarization by Adipose Tissue-Derived Mesenchymal Stem Cells. J Immunol Res (2019) 2019:7059680. 10.1155/2019/7059680 31321244 PMC6607735

[123] Stojanovic S Najman S. The Effect of Conditioned Media of Stem Cells Derived From Lipoma and Adipose Tissue on Macrophages’ Response and Wound Healing in Indirect Co-Culture System In Vitro. Int J Mol Sci (2019) 20(7):1671. 10.3390/ijms20071671 PMC6479913 30987193

[124] Kruger MJ Conradie MM Conradie M van de Vyver M. ADSC-Conditioned Media Elicit an Ex Vivo Anti-Inflammatory Macrophage Response. J Mol Endocrinol (2018) 61(4):173–84. 10.1530/JME-18-0078 30038054

[125] Zhao H Shang Q Pan Z Bai Y Li Z Zhang H. Exosomes From Adipose-Derived Stem Cells Attenuate Adipose Inflammation and Obesity Through Polarizing M2 Macrophages and Beiging in White Adipose Tissue. Diabetes (2018) 67(2):235–47. 10.2337/db17-0356 29133512

[126] Cho KS Kang SA Kim SD Mun SJ Yu HS Roh HJ. Dendritic Cells and M2 Macrophage Play an Important Role in Suppression of Th2-mediated Inflammation by Adipose Stem Cells-Derived Extracellular Vesicles. Stem Cell Res (2019) 39:101500. 10.1016/j.scr.2019.101500 31344653

[127] Heo JS Choi Y Kim HO. Adipose-Derived Mesenchymal Stem Cells Promote M2 Macrophage Phenotype Through Exosomes. Stem Cells Int (2019) 2019:7921760. 10.1155/2019/7921760 31781246 PMC6875419

[128] Zhu D Johnson TK Wang Y Thomas M Huynh K Yang Q. Macrophage M2 Polarization Induced by Exosomes From Adipose-Derived Stem Cells Contributes to the Exosomal Proangiogenic Effect on Mouse Ischemic Hindlimb. Stem Cell Res Ther (2020) 11(1):162. 10.1186/s13287-020-01669-9 32321589 PMC7178595

[129] Xu P Xin Y Zhang Z Zou X Xue K Zhang H. Extracellular Vesicles From Adipose-Derived Stem Cells Ameliorate Ultraviolet B-Induced Skin Photoaging by Attenuating Reactive Oxygen Species Production and Inflammation. Stem Cell Res Ther (2020) 11(1):264. 10.1186/s13287-020-01777-6 32611371 PMC7329484

[130] Chen Y Li J Ma B Li N Wang S Sun Z. MSC-Derived Exosomes Promote Recovery From Traumatic Brain Injury Via Microglia/Macrophages in Rat. Aging (Albany NY) (2020) 12(18):18274–96. 10.18632/aging.103692 PMC7585083 32966240

[131] Deng S Zhou X Ge Z Song Y Wang H Liu X. Exosomes From Adipose-Derived Mesenchymal Stem Cells Ameliorate Cardiac Damage After Myocardial Infarction by Activating S1P/SK1/S1PR1 Signaling and Promoting Macrophage M2 Polarization. Int J Biochem Cell Biol (2019) 114:105564. 10.1016/j.biocel.2019.105564 31276786

[132] Mou S Zhou M Li Y Wang J Yuan Q Xiao P. Extracellular Vesicles From Human Adipose-Derived Stem Cells for the Improvement of Angiogenesis and Fat-Grafting Application. Plast Reconstr Surg (2019) 144(4):869–80. 10.1097/PRS.0000000000006046 31568294

[133] He Q Wang L Zhao R Yan F Sha S Cui C. Mesenchymal Stem Cell-Derived Exosomes Exert Ameliorative Effects in Type 2 Diabetes by Improving Hepatic Glucose and Lipid Metabolism Via Enhancing Autophagy. Stem Cell Res Ther (2020) 11(1):223. 10.1186/s13287-020-01731-6 32513303 PMC7278170

[134] Jin J Shi Y Gong J Zhao L Li Y He Q. Exosome Secreted From Adipose-Derived Stem Cells Attenuates Diabetic Nephropathy by Promoting Autophagy Flux and Inhibiting Apoptosis in Podocyte. Stem Cell Res Ther (2019) 10(1):95. 10.1186/s13287-019-1177-1 30876481 PMC6419838

[135] Chen F Zhang H Wang Z Ding W Zeng Q Liu W. Adipose-Derived Stem Cell-Derived Exosomes Ameliorate Erectile Dysfunction in a Rat Model of Type 2 Diabetes. J Sex Med (2017) 14(9):1084–94. 10.1016/j.jsxm.2017.07.005 28781215

[136] Gebraad A Kornilov R Kaur S Miettinen S Haimi S Peltoniemi H. Monocyte-Derived Extracellular Vesicles Stimulate Cytokine Secretion and Gene Expression of Matrix Metalloproteinases by Mesenchymal Stem/Stromal Cells. FEBS J (2018) 285(12):2337–59. 10.1111/febs.14485 29732732

